# A Systematic Review of Effects of Vitamin E on the Cardiovascular System

**DOI:** 10.7759/cureus.15616

**Published:** 2021-06-12

**Authors:** Sunil Shah, Yasir Shiekh, Jannel A Lawrence, Francis Ezekwueme, Mohammad Alam, Saru Kunwar, Domonick K Gordon

**Affiliations:** 1 Internal Medicine, California Institute of Behavioral Neurosciences & Psychology, Fairfield, USA; 2 Internal Medicine, Mulee Regional Hospital/Ministry of Health, Muli, MDV; 3 Emergency Medicine, California Institute of Behavioral Neurosciences & Psychology, Fairfield, USA; 4 Emergency Medicine, Hamad General Hospital, Doha, QAT; 5 Pediatrics, California Institute of Behavioral Neurosciences & Psychology, Fairfield, USA; 6 Neonatal Intermediate Care Unit, Kanti Childrens Hospital, Kathmandu, NPL; 7 Internal Medicine, Scarborough General Hospital, Scarborough, TTO

**Keywords:** vitamin e, antioxidants, cvd, cardiovascular risks, alpha tocopherol

## Abstract

Vitamin E is a fat-soluble vitamin and an antioxidant that prevents the peroxidation of lipid in vitro. The antioxidant role of vitamin E in preventing adverse cardiovascular outcomes is controversial as some studies support it, while others reject it. Therefore, this review aims to determine whether there is an association between vitamin E and cardiovascular diseases (CVDs). An electronic search was done to find out relevant articles. Papers were shortlisted after the initial title and abstract screen. A full-text study was done, and inclusion and exclusion criteria were applied before the quality assessment of each paper was done. Only high-quality papers were selected for analysis. Full-text articles of the last ten years were included, while non-English articles, gray literature, and animal studies were excluded. The majority of the papers, including 75% of the total population in this review, suggested no role of vitamin E in preventing CVD and CVD mortality. Some studies also suggested that a high level of vitamin E can be associated with adverse cardiovascular outcomes. Thus, one should be prudent about taking vitamin E supplementation for cardiovascular risk prevention.

## Introduction and background

With the increase in advancement in medicine and the health care delivery system, the major cause of morbidity and mortality of the human population is progressively shifting from infectious diseases to non-communicable diseases. Among various important non-communicable diseases, cardiovascular diseases (CVDs) are the major causes of morbidity and mortality. The total deaths due to CVD in 2016 were nearly one-third of all deaths worldwide [1]. In 2017, CVD led to over 330 million years of life lost and 35.6 million years of life lived with disability worldwide [2,3]. The total number of deaths due to CVD in 2017 was over 17 million, and more than 23 million deaths are expected to occur in 2030 across the globe if this rate continues [4].

Vitamin E, one of the fat-soluble vitamins and antioxidants, prevents lipid peroxidation in vitro [5]. Lipid peroxidation is an important initial event in atherosclerosis development and progression, which plays a vital role in developing CVDs [6]. Vitamin E has eight isomers, among which alpha-tocopherol is one of the important forms [5]. Alpha-tocopherol acts as an antioxidant by supplying hydrogen atoms to other radicals that convert the radicals into non-radical products and convert themselves into tocopheroxyl radicals. Tocopheroxyl radical reacts with self or other radicals to form inactive products, thus, breaking the chain of lipid peroxidation reaction.

Some examples of antioxidants are vitamin C, vitamin A, carotenoids, and selenium. Approximately 28%-30% of U.S. (United States of America) adults use supplements that contain various antioxidants [6]. Alpha-tocopherol increases significantly with vitamin E intake and has been postulated to be associated with better cardiovascular health due to antioxidant and anti-atherogenic properties in several studies [7-9].

Some studies have shown the positive effect of vitamin E supplementation against CVD [7-9], while some studies have failed to show its protective role, and few studies have shown possible harmful health effects [10,11]. If there is a significant positive effect of vitamin E on CVDs, supplementation can be done in daily foods to improve the overall cardiac health of the population. However, finding no relation between vitamin E and CVDs would help decrease the unnecessary intake of vitamin E supplementation and possibly prevent its adverse health effect, if there is any. Dietary intake of fruits and vegetables (a rich source of alpha-tocopherol and other antioxidants) is supposed to predict better general health and cardiovascular outcome; however, the role of vitamin E in preventing people from CVD might be due to the combination of various antioxidants and nutrients contained in food. In this systematic review, we attempt to determine if there is any significant association of vitamin E with CVD and CVD mortality.

## Review

Methods

Search Strategy and Selection of Studies

We followed Preferred Reporting Items for Systematic Reviews and Meta-Analyses (PRISMA) as a reporting guideline for this review article. PubMed Central and Medline were used as two databases, and study selection was done by two reviewers independently from February 28 to March 8, 2021. Regular keywords used to collect studies were “Vitamin E AND Cardiovascular Disease,” “Vitamin E AND CVD,” “Tocopherols AND Cardiovascular disease,” and “Tocopherols AND CVD.” MeSH keywords used were ("Vitamin E"[Mesh]) AND "Cardiovascular Diseases"[Mesh]. Only articles that were published in the last 10 years were collected. Initially, studies were selected according to the title's relevance, followed by abstract review using inclusion and exclusion criteria.

Furthermore, duplicates were removed and two assessors independently reviewed full texts, and the final number of studies was determined after two assessors did quality assessment checks. Quality assessment tools used were; Cochrane Bias Assessment Tool for randomized control trial, Amstar 2 Checklist for systemic review, and Newcastle-Ottawa Quality Assessment Scale for observational studies. Only high-quality studies determined by a score of greater than seven and a low risk of bias during the quality assessment were included. The regular keywords used during data search and the total number of articles for each keyword are shown in Table 1.

**Table 1:** Regular Keywords Used for Data Search CVD: Cardiovascular Disease

Regular keywords	Database used	Number of papers
Vitamin E AND Cardiovascular Disease	PubMed	266
Vitamin E AND CVD	PubMed	42
Tocopherols AND Cardiovascular disease	PubMed	108
Tocopherols AND CVD	PubMed	17

The MeSH keywords used during data search and the total number of articles for each keyword are shown in Table 2.

**Table 2:** MeSH Keywords Used for Data Search

MeSH Keywords	Database used	Number of Papers
("Vitamin E"[Mesh]) AND "Cardiovascular Diseases"[Mesh]	PubMed	134

Criteria for Study Selection

Inclusion criteria: (1) All publications in the last 10 years (from January 2011 to January 2021). (2) Articles in the English language. (3) Study population aged >18 years. (4) All types of articles from any country.

Exclusion criteria: (1) All publications older than 10 years (before January 2011). (2) Articles not in the English language. (3) Gray literature. (4) Study population aged <18 years.

Results

Search Results

The electronic database PubMed yielded 567 articles in the initial screening. Two hundred seventy-six articles remained after the removal of duplicates. After screening the title and abstract, 212 unrelated articles were removed, leaving 64 articles for full-text evaluation. Following a full-text study and applying inclusion and exclusion criteria, 38 articles remained that underwent quality assessment.

Study Quality and Characteristics

Out of 38 studies, 25 studies were considered high-quality papers for analysis after quality assessment. Among 25 studies, three were cross-sectional studies, five were case-control studies, 11 were cohort studies, and six were randomized control trials.

**Figure 1:**
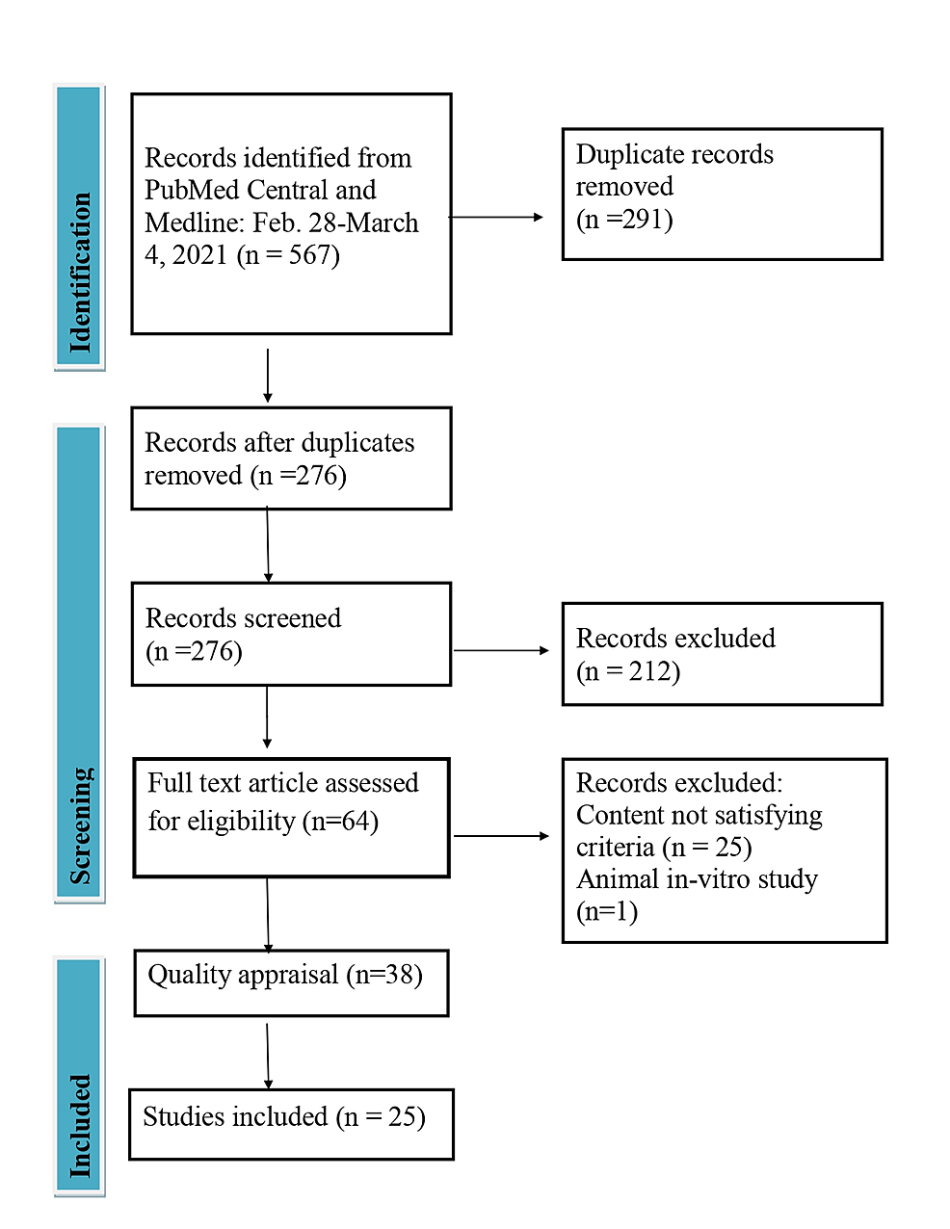
PRISMA Flow Diagram Showing Data Extraction Process

Figure 1 summarizes the data search strategy and selection process.

 

Only high-quality papers were included in the study. The randomized control trial was assessed by Cochrane Risk-of-Bias Tool for Randomized Trials (RoB 2). Only randomized trials with a low risk of bias were included in the study. The quality assessment tools used for cross-sectional [12-14], cohort [15-25], and case-control studies [26-30] were the Newcastle-Ottawa Quality Assessment Tool for cross-sectional, cohort, and case-control studies, respectively. A score of greater than or equal to seven stars during the individual quality assessment, as shown in Table 3, was included in the study.

**Table 3:** Newcastle-Ottawa Quality Assessment of Cross-sectional, Cohort and Case-control Studies max. = maximum

Newcastle-Ottawa Quality Assessment				
Newcastle-Ottawa Quality Assessment of Cross-sectional Studies				
Studies	Selection (max. 5 stars)	Comparability (max. 2 stars)	Outcome (max. 3 stars)	Total Score (max. 10)
Minotti et al. [12]	3	2	3	8
Kuwabara et al. [13]	4	2	3	9
Yildiran et al. [14]	3	2	3	8
Newcastle-Ottawa Quality Assessment of Cohort Studies				
Studies	Selection (max. 4 stars)	Comparability (max. 2 stars)	Outcome (max. 3 stars)	Total Score (max. 9)
Prentice et al. [15]	3	2	2	7
Huang et al. [16]	3	2	3	8
Lee et al. [17]	4	2	3	9
Eshak et al. [18]	4	2	3	9
Zhao et al. [19]	4	2	3	9
Stepaniak et al. [20]	4	2	2	8
Cangemi et al. [21]	3	2	2	7
Wannamethe et al. [22]	4	2	3	9
Espe et al. [23]	3	2	3	8
Goyal et al. [24]	3	2	3	8
Ferro et al. [25]	3	2	2	7
Newcastle-Ottawa Quality Assessment of Case-control Studies				
Llopis-González et al. [26]	4	2	2	8
Godala et al. [27]	2	2	3	7
Xu et al. [28]	2	2	3	7
Naidoo et al. [29]	4	2	2	8
Nagao [30]	4	2	2	8

Actual Result

The total population included in this study was 360,840, among which 63.54% were male and 36.46% were female. Three studies were done in the U.S., and three were from China. Italy and Japan also contributed three studies each, while two studies were done in Australia. The remaining ten articles were obtained from ten individual countries. Two studies included only women, and four included only the male population. Both males and females were included in the remaining articles. The cardiovascular conditions assessed in this review were overall cardiovascular outcome and CVD mortality, hypertension and blood pressure variation, vascular endothelial function, heart failure, myocardial infarction, coronary artery diseases, recurrence of atrial fibrillation, ischemic stroke, and low-density lipoprotein peroxidation. Two studies included cardiovascular outcomes in type 2 diabetes mellitus patients, and two studies included cardiovascular outcomes in end-stage renal disease patients.

Discussion

Many people use vitamin E supplements worldwide as it acts as an antioxidant and is supposed to prevent cardiovascular risks; for example, 28%-30% of U.S. adults use supplements containing antioxidants such as vitamin E [6]. However, whether the antioxidant role of vitamin E is beneficial to prevent adverse cardiac outcomes or not is still controversial. Some studies support the thesis that the antioxidant role of vitamin E is beneficial in preventing cardiac disease and overall cardiac mortality [7-9]. However, some studies are against this thesis [10,11]. Thus, it is important to solve the question- Is the antioxidant property of vitamin E truly beneficial to protect CVD and CVD mortality?

Vitamin E as an Antioxidant

**Figure 2:**
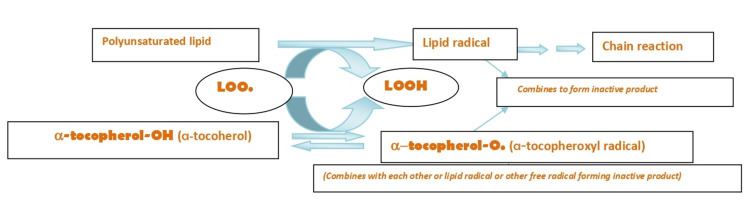
Reaction Showing Peroxidation of Lipid and Vitamin E as an Antioxidant LOO = lipid-peroxyl radical, LOOH = lipid hydroperoxide

Alpha-tocopherol is a major isomer of vitamin E in vivo, which acts as an antioxidant by scavenging the free radicals of unsaturated lipids [31]. Lipid peroxidation, which has an important role in atherosclerosis and CVDs, has three stages; initiation, propagation, and termination [32]. Initially, carbon-centered lipid radical (alkyl radical) is produced from unsaturated lipid. This reaction is catalyzed by heat, light, and transition metals [32]. The alkyl radical then reacts with oxygen at a very high rate forming a peroxyl radical that can further attack another polyunsaturated lipid molecule propagating the chain reaction. The chain reaction continues until the peroxyl radical meets another radical and forms an inactive product (termination). Alpha-tocopherol transfers hydrogen atom to lipid-free radical (peroxyl, alkoxyl, and carbon-centered radical), giving rise to a non-radical lipid product and an alpha-tocopheroxyl radical. The alpha-tocopheroxyl radicals self-combine or react with other free radicals to form inactive products, thus, terminating the chain propagation. The summary of the major antioxidant property of alpha-tocopherol (a major isoform of vitamin E) is depicted in Figure 2 [32].

Antioxidant Role of Vitamin E in Preventing CVD and CVD Mortality

A total of 25 studies were included in this review. Of 25 studies, 13 studies showed no association of vitamin E with CVDs. Zhao et al. 2017, a cohort study that included 134,358 participants, concluded that vitamin E is not associated with CVD mortality or total mortality in a long follow-up period of 8.2 years in males and 14.2 years in females; however, vitamin C and carotene were inversely associated with CVD mortality and overall mortality [19]. Similarly, Nagao et al., a case-control study involving 38,158 participants, showed no significant association between coronary heart disease and serum alpha- or gamma-tocopherol level [30]. Two studies were done in women only, Prentice et al. 2019 and Chae et al. 2012 [15, 33]. Both of these studies concluded that the role of vitamin E is not significant in preventing CVD and CVD mortality. However, Eshak et al. 2017, a cohort study involving 58,696 participants, showed that a higher intake of vitamin E was associated with a lower CVD mortality risk in females but not in men in the median follow-up period of 19.3 years [18].

Four studies included the male population only. Huang et al. 2019, a cohort study which included 29,133 men, showed the decreased cardiovascular mortality and overall mortality in participants with higher vitamin E level during 30 years of follow up [16]. Minotti et al. 2017, a cross-sectional study that included 615 males, concluded that higher serum vitamin E level was associated with decreased impaired flow-mediated dilation of the brachial artery [12]. Wannamethe et al. 2013, a cohort study including 3,919 men, showed that vitamin E is not significantly associated with decreased risk of heart failure [23]. Yildiran et al. 2011, a cross-sectional study including 66 men, concluded that lower serum vitamin E level is associated with congestive heart diseases [15]. The overall characteristics of the articles included in this review are shown in Table 4.

**Table 4:** Summary of the Articles Included in the Review AMI: Acute myocardial infarction; B.P.: Blood pressure; CHD: Chronic heart disease; CVD: Cardiovascular diseases; D.M.: Diabetes Mellitus; ESRD: End-stage renal disease; F: Female; FMD: Flow-mediated Dilation; HTN: Hypertension; LDL: Low-density lipoprotein; M: Male; RCT: Randomized Control Trial; TRF: Tocotrienol rich fraction; U.K.: United Kingdom; U.S.: United States

Author	Year	Location	Study type	Population	Results/Conclusion
Prentice et al. [15]	2019	U.S.	Cohort	3780 all female (F)	No significant association of vitamin E in decreasing CVD risk.
Huang et al. [16]	2019	Finland	Cohort	29133 all male (M)	Men with higher vitamin E status had significantly lower overall mortality and cause-specific mortality due to CVD found during 30 years of follow up independent of several other mortality risk factors.
Lee et al. [17]	2018	China	Cohort	875 (M=456, F=419)	Increased dietary intake of vitamin E was significantly associated with decreased risk of long term cardiovascular outcome during 22 years of follow up in people with age 44.7+/-11.5 years
Eshak et al. [18]	2017	Japan	Cohort	58696 (M=23099, F=35597)	Higher intake of vitamin E or vitamin D, vitamin K was associated with lower cardiovascular mortality risk in women but not in men during the median follow up of 19.3 years.
Alshiek et al. [34]	2017	Israel	RCT	20 (M=9, F=11)	Significant improvement of vascular function following eight weeks of treatment with vitamin E in type 2 D.M. patients with hp2-2 gene who were of age >55 years.
Minotti et al. [12]	2017	Italy	Cross-sectional	615 all M	Vitamin E to the total intake of calories was inversely associated with impaired flow-mediated dilation (FMD) of the brachial artery. The impaired FMD is a marker of early arterial vascular dysfunction, which is an important feature of atherosclerosis.
Zhao et al. [19]	2017	China	Cohort	134358 (M=59739)	Total carotene and vitamin C level was inversely associated with CVD mortality and all-cause mortality; however, vitamin E was not associated with either of them during a long follow-up period (8.2 years for male, 14.2 years for female).
Godala et al. [27]	2017	Poland	Case-control	191 (M=101 F=90)	Serum vitamin E level together with vitamin A, C, and D was significantly low in people aged 30 to 65 with metabolic syndrome.
Stepaniak et al. [20]	2016	Russia, Poland, Czech	Cohort	26993 (M=12642 F=14351)	No clear association between vitamin E intake and CVD/mortality. No significant protective effect of vitamin E or vitamin C, beta carotene against CVD.
Stone house et al. [35]	2016	South Australia	RCT	87 (M=54, F=33)	Eight weeks supplementation of palm tocotrienol TRF-80 (420 mg/day tocopherol+132 mg/day tocotrienol) significantly increased the level of serum vitamin E; however, it did not significantly affect vascular endothelial function and other markers of CVD.
Llopis- González et al. [26]	2015	Spain	Case-control	1514 (M=752, F=762)	No significant association of hypertension with the intake of vitamin E in people of age >40 years
Kuwabara et al. [13]	2014	Japan	Cross-sectional	3507 (M=1405, F=2102)	Higher vitamin E intake was associated with a low prevalence of HTN significantly in people of age >40 years
Hodgson et al. [36]	2014	Australia	RCT	55 (M=41, F=14)	Vitamin E was not associated significantly with day time or night time B.P. variation in Type2 D.M. patients of mean age 61.3 years
Xu et al. [28]	2014	China	Case-control	60 (M=26, F=34)	An acute increase in blood sugar can damage the vascular endothelial function, especially in hypertensive patients. This effect was reversed significantly by a high dose of vitamin E and/or vitamin C.
Cangemi et al. [21]	2013	Italy	Cohort	1012 (M=55%, F=45%)	Low serum vitamin E was associated with an increased number of cardiovascular events such as myocardial infarction, ischemic stroke, and cardiovascular death in a patient with non-valvular atrial fibrillation.
Wannamethe et al. [22]	2013	U.K.	Cohort	3919 All M	Serum vitamin C but not vitamin E was found to be significantly associated with the reduced risk of heart failure in men with or without preexisting MI.
Otero-Losada et al. [37]	2013	Argentina	RCT	112 (M=51, F=61)	Serum alpha-tocopherol level was increased only in people with pretreatment alpha-tocopherol less than that of recommended serum level otherwise, there was no effect of supplementation of 400mg alpha-tocopherol daily for two months. No association between atherosclerotic cardiovascular disease and supplementation of vitamin E was found.
Espe et al. [23]	2013	Germany	Cohort	1046 (M=565, F=481)	Serum alpha-tocopherol level was not significantly associated with cardiovascular outcome and all-cause mortality in diabetic hemodialysis patients.
Goyal et al. [24]	2013	U.S.	Cohort	16008 (M=7510 F=8498)	Lower or higher serum level of vitamin E was significantly associated with increased risk of all-cause mortality. However, it was not associated with cardiovascular disease/mortality.
Baldi et al. [38]	2012	Austria	RCT	37	Vitamin E supplementation increased the resistance of LDL to oxidation after hemodialysis in 18 patients on chronic hemodialysis due to ESRD despite the fact that each dialysis session acutely increases LDL oxidizability.
Naidoo et al. [29]	2012	Singapore	Case-control	699 (M=456, F=243)	Alpha and gamma-tocopherol were not associated with an increased or decreased risk of acute myocardial infarction (AMI). However, delta-tocopherol was significantly associated with an increased risk of AMI.
Chae et al. [33]	2012	U.S.	RCT	39815 all F	Vitamin E was not associated with the overall risk of heart failure in females aged >45 years, who were healthy at baseline, on 10.2 years of median follow-up time.
Nagao et al. [30]	2012	Japan	Case-control	38158 (M=13382 F=24776)	No significant association was found between alpha or gamma-tocopherol and coronary heart disease. Serum alpha-tocopherol was associated with decreased total and hemorrhagic stroke mortality in females, while gamma-tocopherol was associated with increased hemorrhagic stroke mortality in females but lower ischemic stroke mortality in men.
Ferro et al. [25]	2012	Italy	Cohort	144 (M=83, F=61)	Low serum vitamin E level was associated with increased risk of atrial fibrillation recurrence in patients who underwent cardioversion.
Yildiran et al. [14]	2011	Turkey	Cross-sectional	66 all M	Vitamin E intake in 35 men with CHD was lower than in 31 men without CHD (p<0.05) aged between 40-65 years.

Among 25 studies included in this review, most of the studies (i.e., 13 out of 25) failed to show any significant association regarding the role of vitamin E in preventing adverse cardiovascular outcomes. Twelve of them concluded that there is a significant association of vitamin E in preventing CVDs. Considering that a large number of participants were included in this review (360,840), it is worth noting that 12 studies, including only 93,741 participants, showed the association of vitamin E in preventing CVDs. To make it easier to interpret the data that we got in these twelve studies, studies including only 25.9 % of participants compared to the total population in this review supported the association of vitamin E with CVD/mortality. Those studies were; five cohort studies, three case-control studies, three cross-sectional studies, and two randomized control trials.

From this study, we may conclude that vitamin E has no role in preventing CVDs. Thirteen out of twenty-five studies, including 267,099 participants, almost 75% of the total participants, rejected the hypothesis that vitamin E has a role in preventing CVD and cardiovascular mortality. This result tends to reject the hypothesis that vitamin E has a role in decreasing cardiovascular risks. According to Otero-Losada et al. 2013, even after supplementary vitamin E intake, serum vitamin E levels increased only in the participants who had lower than recommended initial vitamin E levels [37]. The studies that rejected the association between vitamin E and CVD were; six cohort studies, three case-control studies, and four randomized control trials. Some studies showed that higher vitamin E levels could increase myocardial infarction and stroke risk [29,30].

Limitations

One of the possible limitations of this study includes the limited availability of review articles, case-control, and cohort studies. In addition, non-English papers and grey literature were excluded. Also, a full-text article, if not available, was excluded from this study. No appropriate nutrient data was available, and baseline circulating vitamin E was also unavailable in several studies. Furthermore, the result may have been affected by confounding factors in the studies included in this review, such as physical activity and alcohol intake.

## Conclusions

We attempted to determine the protective role of vitamin E, if there is any, against the adverse cardiovascular outcomes in this paper. According to this review, we concluded that there is no significant correlation between vitamin E and cardiovascular risks. Most of the studies rejected the thesis that higher vitamin E intake helps prevent CVDs. Some studies even showed that higher serum vitamin E levels could be detrimental to health. Thus, vitamin E intake as a supplement to protect against CVDs is not a good practice. One should be more cautious while taking or prescribing vitamin E supplementation. In addition, a large-scale study is needed to be done to find out whether the high serum level of vitamin E is associated with adverse health outcomes or not.
